# 

**Published:** 1898-04-16

**Authors:** 


					--JmvJI " ine standard of highctt purity."- Lancet. April 16th, 1898.
/ CADBURY'S ^ ** CADBURY'S
cocoa Inll lir cocoa
ABSOLUTELY PURE. C?> ?3 NO DRUGS OR ALKALIES USED.
No. 603. Vol. XXIV.
[3SSH Saturday,
April 16th, 1898.
[ Trp.n4o J PRICE TWOPENCE.

				

